# Multidimensional quality evaluation and traceability study of Fritillariae Cirrhosae Bulbus from different sources

**DOI:** 10.3389/fpls.2025.1648434

**Published:** 2025-09-11

**Authors:** Guiqi Han, Luming Qi, Dongmei He, Weihang Xue, Wenshang Li, Hai Wang, Zhuyun Yan

**Affiliations:** ^1^ State Key Laboratory of Characteristic Chinese Medicine Resources in Southwest China, Chengdu University of Traditional Chinese Medicine, Chengdu, China; ^2^ School of Pharmacy, Chengdu University of Traditional Chinese Medicine, Chengdu, China; ^3^ School of Medical Technology, Chengdu University of Traditional Chinese Medicine, Chengdu, China; ^4^ School of Health Preservation and Rehabilitation, Chengdu University of Traditional Chinese Medicine, Chengdu, Sichuan, China

**Keywords:** Fritillariae Cirrhosae Bulbus, metabolomics, alkaloids, mineral elements, traceability model

## Abstract

The quality of “Fritillariae Cirrhosae Bulbus (FCB)” is influenced by its geographical origin and cultivation management. Characterizing quality differences among FCB from different sources through multidimensional analysis and establishing an accurate traceability model represent critical approaches to ensure FCB medicinal material quality. This study integrated untargeted metabolomics, alkaloid quantification, mineral nutritional element analysis, and hyperspectral imaging features to systematically reveal metabolic and compositional variations in FCB from different sources, while constructing a deep learning-based traceability model. Untargeted analysis identified significant differences in metabolite levels across FCB sources, with Kyoto Encyclopedia of Genes and Genomes (KEGG) pathway analysis revealing that these differential metabolites were primarily enriched in 23 pathways. Targeted alkaloid quantification demonstrated that field-collected wild specimens from Seka township (designated SK-FC) accumulated higher levels of peimisine, imperialine, and peiminine, whereas tissue-cultured regenerants from Bamei town (designated BM-TC) exhibited elevated peimine content, indicating that geographical environments and cultivation practices regulate alkaloid biosynthesis. Mineral nutritional element analysis showed that BM-TC samples had the highest elemental accumulation, likely linked to nutrient-rich culture media, while field-collected wild specimens from Chuanzhusi town (designated CZS-FC) and Anhong township artificial cultivated accessions (designated AH-AC) preferentially accumulated Al/Fe/Mn/Na and K/Mg/Zn/Cu, respectively. Most elements showed positive correlations with peiminine and peimine levels but negative correlations with peimisine and imperialine. The Residual Network (ResNet) deep learning model, constructed using hyperspectral-derived three-dimensional correlation spectroscopy (3DCOS) images, achieved 100% testing/validation accuracy and 86.67% external validation accuracy, outperforming traditional partial least squares discriminant analysis (PLS-DA) models in traceability efficacy and providing an efficient method for precise origin identification of FCB. This research establishes theoretical foundations for multidimensional quality evaluation and traceability of FCB, offering fundamental support for further development and utilization of FCB resources.

## 1 Introduction

Fritillariae Cirrhosae Bulbus (FCB), the dried bulbs of several species belonging to the *Fritillaria* genus (Liliaceae family), was first documented in the *Shennong’s Classic of Materia Medica* in China. This document marks the beginning of its medicinal history that has spanned over 2,000 years (Xin et al., 2014). A multitude of studies have demonstrated that FCB possesses a variety of therapeutic properties, including antitussive, expectorant, anti-inflammatory, anti-asthmatic, sputum-eliminating, anticancer, acute lung injury-alleviating, and anti-fibrotic effects (Wu et al., 2022; Wang et al., 2021). FCB is the primary raw material for over 210 Chinese patent medicines, and its annual demand can reach up to 5,000 tons. However, the supply of FCB is often inadequate to meet this demand (Cunningham et al., 2018). The six FCB source species listed in the *Chinese Pharmacopoeia* predominantly inhabit high-altitude alpine regions (3,000-4,500 m above sea level) with restricted, suitable habitats, low natural reproductive capacity, and poor regeneration of wild resources. Environmental degradation and excessive harvesting have caused sharp declines in wild populations (Song et al., 2021; Mathela et al., 2021). Consequently, all wild FCB source species are classified as “Class II State-Protected Plants” in China, thereby prohibiting indiscriminate collection (Zhao et al., 2025). Domestication and cultivation of FCB have achieved significant progress, with cultivated varieties now constituting the primary supply. Cultivation bases established in suitable regions (including Sichuan, Yunnan, Xizang, Gansu, and Qinghai) demonstrate that variations in ecological environments and cultivation methods impact the quality and efficacy of FCB. As the public’s attention to traditional Chinese medicine (TCM) grows, there is an increased emphasis on quality consistency and the ability to trace the origins of these products. Concurrently, improvements in regional logistics systems have facilitated the mixing of medicinal materials from diverse sources, making multidimensional quality evaluation and traceability of FCB imperative (Wang et al., 2024a).

The intricate composition and substantial quality variations inherent in TCM present challenges to conventional quality control methodologies, resulting in limitations in precision and applicability (Ren et al., 2024). Conventional single-method detection strategies are inadequate in capturing the complexity of TCM. Therefore, the integration of multidimensional evaluation approaches to enhance the accuracy and reliability of TCM quality assessment, as well as the establishment of rapid, precise traceability models, is essential for advancing the sustainable development of the TCM industry (Xiao et al., 2024; Nie et al., 2019).The rapid advancement of analytical technologies has provided robust tools for the quality evaluation of FCB from diverse sources. Untargeted metabolomics is a technique that detects all ionizable metabolites within a specific mass range (Yao et al., 2019). In contrast, targeted metabolomics is a method that allows for the precise quantification of specific metabolite classes (Zhou and Yin, 2016). Elemental analysis is a technique that is used to decipher mineral element profiles in FCB (Ågren and Weih, 2020). The integration of these technologies enables the provision of scientific evidence and data, facilitating a comprehensive evaluation of the overall quality of active components and mineral elements in FCB.

Spectroscopic technology is a non-destructive analytical method. It enables rapid detection and real-time analysis without requiring sample pretreatment (Armenta et al., 2008). The integration of spectral data with deep learning algorithms facilitates the construction of origin-discrimination models, thereby facilitating precise traceability (Jiang et al., 2024; Zhao et al., 2024; Wang Y. Y. et al., 2022). Residual Network (ResNet), an exceptional convolutional neural network framework, addresses common training challenges in deep networks, such as vanishing and exploding gradients (Dong et al., 2021; Lecun et al., 2015). Chen et al. (2022) developed a methodology for generating two-dimensional correlation spectroscopy (2DCOS) images from near-infrared (NIR) spectral data of *Centella asiatica*. They then integrated these images with ResNet, achieving 100% accuracy geographically. In addition, Li et al. (2025b) developed a ResNet-based identification model using three-dimensional correlation spectroscopy (3DCOS) images derived from *Gastrodia elata* NIR spectral data. The model demonstrated a good level of accuracy, achieving 100% accuracy on the test set and maintaining 95.45% accuracy on the external validation. These results demonstrate the excellent performance of multidimensional spectral fusion in conjunction with deep learning in detecting minute variations.

This study applied a multidimensional evaluation framework of “metabolism-component-environment” to systematically profile global metabolic profiles in FCB. Untargeted metabolomics was used to identify metabolic variations, while targeted metabolomics was employed to quantify major alkaloids. Mineral element analysis was utilized to assess environmental influences on metabolic pathways. Subsequently, we developed a synchronous 3DCOS-ResNet model using non-destructive hyperspectral imaging to differentiate FCB geographical origins. Collectively, these multidimensional chemical analyses identify variations in FCB quality and establish traceability models, providing novel approaches for quality evaluation and origin identification.

## 2 Materials and methods

### 2.1 Sample collection

**Figure 1:**
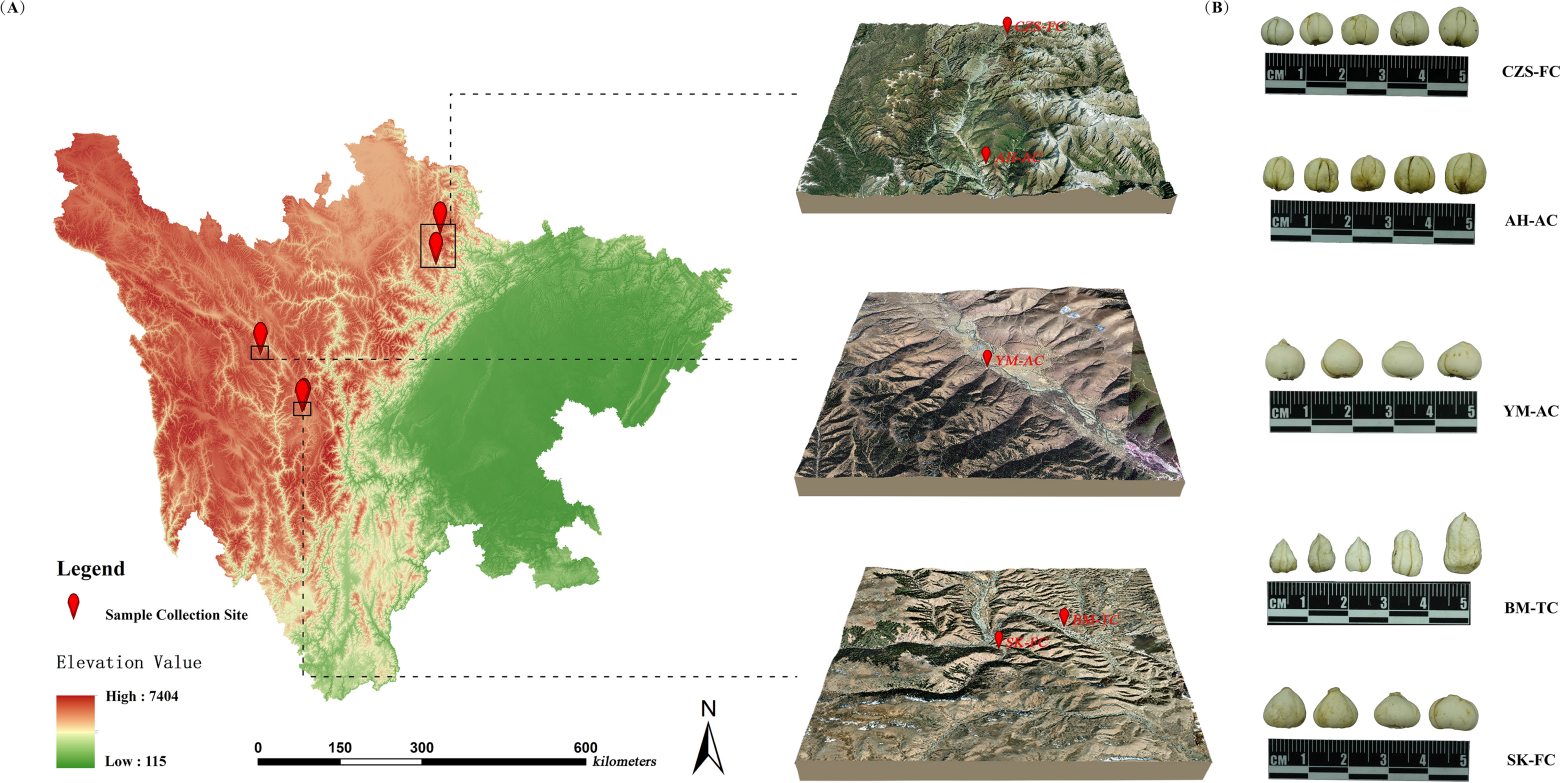
Information of FCB samples. **(A)** Overview of research areas; **(B)** FCB from different sources.

A total of 90 FCB samples were collected from Sichuan Province, China. These samples were morphologically and genetically identified as *Fritillaria cirrhosa* D.Don (Family, Liliaceae) by Professor Zhuyun Yan of the Chengdu University of Traditional Chinese Medicine and classified into five distinct source categories. The sample origins and identification codes are detailed in **Table 1**, and the geographical distribution is illustrated in **Figure 1**. After collection, the FCB samples were washed, air-dried naturally to constant weight, and ground into fine powder. The powder was sieved through a 100-mesh sieve and subsequently stored in sealed bags for subsequent analyses.

**Table 1:** The source of FCB.

Sample iD	Provenance		Geographic coordinates			
	Production area	Agronomic practice	Longitude	Latitude	Altitude (m)	Number of specimens
AH-AC	Anhong Township, Songpan County, Aba Prefecture	Artificial cultivation	103.647596 E	32.539952 N	3216	18
CZS-FC	Chuanzhusi Town, Songpan County, Aba Prefecture	Field collection	103.715100 E	32.961250 N	3387	18
BM-TC	Bamei Town, Daofu County, Ganzi Prefecture	Tissue culture regeneration	101.473850 E	30.493240 N	3406	18
SK-FC	Seka Township, Daofu County, Ganzi Prefecture	Field collection	101.447594 E	30.476054 N	3584	18
YM-AC	Yimu Township, Luhuo County, Ganzi Prefecture	Artificial cultivation	100.760640 E	31.283110 N	3125	18

### 2.2 Reagents and materials

Reference standards (HPLC grade) of peimisine (CAS: 19773-24-1), imperialine (CAS: 61825-98-7), peiminine (CAS: 18059-10-4), and peimine (CAS: 23496-41-5) were supplied by Chengdu Alpha Biotech Co., Ltd. (China). Single-element stock solutions (Na: 23C001-1; K: 23C020; P: 238051; Ca: 238006-2) and the 24-element mixed standard stock solution (245019-1) were certified reference materials that had been accredited at a national level.

Methanol, formic acid, ammonium acetate, and acetonitrile (HPLC grade) were purchased from Thermo Fisher Scientific (USA). Ultrapure water (HPLC grade) was obtained from Merck (Germany). Nitric acid and other analytical grade reagents were procured from Chengdu Kelong Chemical Reagent Co., Ltd. (China).

### 2.3 Untargeted metabolomic profiling

A precisely weighed 0.1 g sample of liquid nitrogen-ground FCB powder was mixed with 500 μL of an 80% methanol aqueous solution. The mixture was vortexed and then incubated on ice for 5 min. It was then centrifuged at 15,000 ×g at 4°C for 20 min. A portion of the resulting supernatant was diluted with water to reduce the methanol concentration to 53%, after which it was centrifuged again under the same conditions (15,000 ×g, 4°C, 20 min). The resulting supernatant was filtered through a 0.22 μm membrane filter and analyzed using Ultra-Performance Liquid Chromatography-Tandem Mass Spectrometry (UPLC-MS/MS) (Want et al., 2013).

The UPLC-MS/MS analysis of sample was conducted on a UHPLC-Q Exactive system equipped with an ACQUITY HSS T3 column (100 mm × 2.1 mm i.d., 1.8 μm; Waters, USA) at Majorbio Bio-Pharm Technology Co. Ltd. (China). The samples were separated on the chromatographic column and the temperature was maintained of 40°C and a mobile phase flow rate of 0.2 mL/min. In positive mode, the mobile phase consisted of 0.1% formic acid (A) and methanol (B), and in negative mode, it consisted of 5 mM ammonium acetate (A) and methanol (B). The initial ratio was 98% A and 2% B, and the following gradient program was used: 0-3 min 98%-0% A; 3-10.1 min, 0%-98% A; 10.1-12 min, 98%-98% A. The injection volume was 2 μL.

The UHPLC system was coupled to a UHPLC-Q Exactive system Mass Spectrometerequipped with an electrospray ionization (ESI) source operating in positive mode and negativemode. The system under the following parameters: Scanning range: 100-1500 m/z; spray voltage: 3.5 kV; sheath gas flow rate: 35 psi; auxiliary gas flow rate: 10 L/min; ion transport tube temperature: 320°C; iontophoresis RF level: 60; auxiliary gas heater temperature: 350°C; polarity: positive, negative; MS/MS secondary scanning was data-dependent scanning.

### 2.4 Targeted alkaloid quantification

A precisely weighed 0.1 g sample of FCB powder was precisely weighed out mixed with 1.2 mL of a 70% methanol aqueous solution. This mixture was vortexed for 30 min, repeated six times and then stored in a refrigerator overnight. The mixture was then centrifuged at 15,000 ×g and 4°C for 20 min. A portion of the resulting supernatant was diluted with water to reduce the methanol concentration to 53%. This was then subjected to another round of centrifugation under identical conditions (15,000 ×g, 4°C, 20 min). The resulting supernatant was filtered through a 0.22 μm membrane filter to obtain the FCB extracts.

The UPLC-MS/MS analysis was performed in the following sequence concerning Han’s method (Han et al., 2023) and with appropriate adjustments. Chromatographic separation was performed using a TSQ-Fortis triple quadrupole mass spectrometer (Thermo Fisher, Germany). The column (Hypersil Gold™: 100 mm×2.1 mm, 3 um) temperature was maintained at 35°C with a mobile phase flow rate of 0.3 mL/min. The mobile phase consisted of water containing 0.3% (v/v) formic acid (A) and acetonitrile (B), with an initial composition of 80% A and 20% B. The following gradient program was applied:0-5 min: 80%-60% A. Injection volume: 2 μL (Wang et al., 2025).

The TSQ-Fortis mass spectrometer operated in default mode. The specific parameters are as follows: Ion source type: H-ESI; positive ion: 3500 V; negative ion: 3500 V; sheath gas flow: 35 Arb; auxiliary gas: 15 Arb; purge gas: 1 Arb; ion transport tube temperature: 350°C; vaporizer temperature: 350°C; different sources of FCB extract were analyzed under multiple reaction monitoring mode. Full details of the mass spectrometric parameters for four steroidal alkaloids and comprehensive method validation data are detailed in **Table 2**, **Supplementary Table S1** and **Supplementary Figure S1**.

**Table 2:** MS parameters of 4 alkaloids.

Analyte	Retention time (min)	Parent ion (m/z)	Product ion (m/z)	Collison energy (ev)
Peimisine	2.40	428.40	114.15*, 337.22	29.43
Imperialine	1.89	430.45	138.24*, 412.48	47.67
Peiminine	2.90	430.40	396.38*, 112.08	54,85
Peimine	2.59	434.45	398.46*, 414.39	55.00

### 2.5 Nutritional element analysis

A precisely weighed 0.2 g aliquot of FCB powder was placed into a digestion vessel. After adding 5 mL of nitric acid (HNO_3_), the vessel was capped and allowed to stand for 1 h. The lid was then tightly sealed, and digestion was performed following the microwave digestion system’s standard operating procedures (**Supplementary Table S2**). After cooling, the vessel was slowly opened to release gases. The inner lid was rinsed with a small amount of water, and the vessel was placed on a temperature-controlled hotplate at 80°C for 3 min to remove brown fumes. The digestate was transferred to a 25 mL volumetric flask, and the vessel was rinsed three times with small amounts of water. The rinsates were combined and diluted to the mark with water, followed by thorough mixing. Blank controls were prepared simultaneously.

Standard stock solutions were serially diluted with 5% nitric acid to prepare calibration standards. An internal standard mixture was added to all standard solutions to correct for matrix effects and instrumental drift, ensuring measurement accuracy. Discrepancies between measured and certified values were maintained below 10%, with recovery rates ranging from 95% to 100%.

Mineral elements (K, Na, Mn, Fe, Al, Cu, Mg, Zn) were quantified using inductively coupled plasma mass spectrometry (ICP-MS). Key operating parameters:plasma airflow: 15.0 L/min; auxiliary gas flow: 1.5 L/min; the flow rate of nebulizer was 0.75 L/min. Power: 1200 W.

### 2.6 Hyperspectral data acquisition

Hyperspectral imaging was performed using a handheld HY-6010 imager (Hangzhou Gaopu Imaging Technology Co., Ltd, China) spanning 400-1000 nm with 300 contiguous spectral channels at 5 nm resolution (FWHM), configured with a 55 mm aperture lens, 480 × 485 pixel spatial resolution, dual 100 W tungsten-halogen lamps equipped with hemispherical diffuse reflection domes (Labsphere Inc., USA) positioned symmetrically at 45° to eliminate specular reflection, and a fixed 30 ms exposure time optimized through pre-scan radiometric calibration using 99% Spectralon^®^ standard. Hyperspectral data collection was conducted in a darkroom. Samples were sequentially arranged on the mobile platform of the hyperspectral imaging system. During system operation, the platform moved steadily, and the lens captured and recorded sample image information. To ensure clear and complete images, parameters were repeatedly adjusted, with the final integration time set to 142961 and the frame rate to 7 fps. To mitigate the effects of dark current and unstable light sources, radiometric calibration was performed on the acquired images using a synchronously captured radiometric calibration panel (20% reflectance) via the HHITSYSPEC software (Version 1.9.1). Using the calibrated images, regions of interest (ROIs) were selected for each sample as individual units. Distinct ROIs were labeled with different colors, and the average reflectance spectrum of each sample’s ROI was calculated. Each ROI generated a corresponding spectral curve, which served as the dataset for subsequent analysis.

### 2.7 Construction of partial least squares-discriminant analysis model

PLS-DA is a supervised learning framework that facilitates the visual classification of samples by integrating the dimensionality reduction capability of partial least squares with the classification strategy of discriminant analysis (Qi et al., 2018). The model’s dimensionality reduction strategy is predicated on the supervised extraction of latent variables (LVs), which are iteratively: The primary objective is to maximize the shared variability between X (predictor variables) and Y (class labels). This is achieved by implementing orthogonalization, a process that eliminates redundancy. Additionally, cross-validation is employed to ascertain the optimal dimensionality. This process involves the compression of high-dimensional data into a low-dimensional space, thereby reducing the quantity of features while preserving those that are critical for classification. Consequently, the model attains enhanced classification accuracy and generalization capability for high-dimensional complex data. In this study, the first two LVs were extracted to holistically visualize the differences among FCB from diverse sources (Qi et al., 2024).

### 2.8 ResNet-based traceability modeling

#### 2.8.1 3DCOS image generation

The 3DCOS technique effectively resolves overlapping spectral peaks and enhances discrimination capability by introducing an additional dimension, thereby improving spectral resolution (Liu et al., 2023a). Based on the aforementioned hyperspectral data, 3DCOS images were generated using MATLAB software. These images were calculated according to the discrete generalized 3DCOS algorithm, with the Formulas (1) and (2) as follows (Liu et al., 2023b):


(1) $$S(v)=\{\begin{array}{c} s(v,t_{1})\\ \begin{array}{c} s(v,t_{2})\\ \cdots \end{array}\\ s(v,t_{m}) \end{array}$$

(2) $$\varphi (v_{1},v_{2})=\frac{1}{m-1}S{\left(v_{1}\right)}^{T}S(v_{2})$$

The dynamic spectral intensity at the variable v is expressed as S, t is the external disturbance, *m* is the spectrum measured at equal intervals between the disturbances t, and is the expression of the three-dimensional correlation intensity. The transformed spectra were stored as JPG images with a resolution of 875×656 pixels. The 3DCOS spectra were generated from the hyperspectral dataset of FCB using Matlab R2019b based on the three-dimensional correlation intensity formula.

#### 2.8.2 Model construction

The primary strategy employed by the ResNet model for dimension reduction is the implementation of convolution operations on the input data to generate feature maps. This is followed by the utilization of batch normalization layers to standardize the data, thereby facilitating accelerated model training. The ReLU function is then employed to achieve nonlinear activation. Subsequently, the processed data is entered into three identity blocks and two convolution blocks for feature extraction. The global average pooling method is employed to extract the primary features, thereby reducing the number of parameters. The Softmax function is utilized in the final layer to generate the results (Li et al., 2025a).

These 3DCOS images were subsequently used for ResNet modeling. A 12-layer ResNet image recognition model was constructed using training and testing sets to specifically identify 3DCOS images of FCB from different sources. Key hyperparameters included a weight decay coefficient (λ) of 0.0001 and a learning rate of 0.01. Accuracy curves and cross-entropy loss function curves were generated using Mxboard, with a smoothing parameter set to 0.6 (Li et al., 2025b). In these curves, the x-axis represents epochs, while the y-axis corresponds to Acc and loss value, respectively. Fewer epochs indicate higher modeling efficiency. Accuracy and loss value are critical metrics for evaluating classification performance and convergence. The model’s generalization ability was ultimately assessed using an external validation set. An Acc closer to 1 signifies superior recognition performance, and a loss value closer to 0 indicates better convergence. The present study employed random sampling to allocate the samples into training sets, testing sets, and external validation sets, with 50, 25, and 15 samples, respectively. The utilization of random sampling ensures the mitigation of subjective bias that may be introduced during manual sample selection. This approach facilitates objective evaluation of model performance, prevents overfitting, and enhances model stability and repeatability.

ResNet addresses the vanishing gradient problem in deep network training by introducing residual blocks, enabling the construction of deeper networks simultaneously (Su et al., 2021). Its core architecture consists of residual blocks, each typically containing two or three convolutional layers. Each convolutional layer is followed by batch normalization and a ReLU activation function. Through skip connections, the input is directly added to the convolutional layer outputs to form residual outputs. The Formula (3) is expressed as:


(3) $$y=F(x,W_{i})+x$$

Where *F(x, W_i_)* is the output of the convolution layer, x is the input, and y is the output of the residual block.

The overall architecture comprises an input layer, convolutional layers formed by multiple residual blocks, pooling layers, and fully connected layers. The input layer converts images into feature maps, the convolutional layers progressively extract high-level features, the pooling layers reduce feature map dimensionality, and the fully connected layers ultimately perform classification tasks. By incorporating skip connections, ResNet effectively mitigates vanishing and exploding gradient issues in deep networks, maintaining superior performance even as network depth increases (Wu et al., 2019).

Model evaluation metrics included accuracy (Acc), precision (Pre), sensitivity (Sen), and specificity (Spe), calculated as follow Formulas (4) to (7) (Zheng et al., 2023):


(4) $$Acc=\frac{TP+TN}{TP+FP+TN+FN}$$

(5) $$\mathrm{Pr}e=\frac{TP}{TP+FP}$$

(6) $$Sen=\frac{TP}{TP+FN}$$

(7) $$Spe=\frac{TN}{TN+FP}$$

Among them, TP represents the number of true cases, FP represents the number of false positive cases, TN represents the number of true negative cases, and FN represents the number of false negative cases.

### 2.9 Data processing

#### 2.9.1 Untargeted data processing

Raw data files (.raw) were imported into the Compound Discoverer3.1 library search software for processing. Parameters such as retention time and mass-to-charge ratio (m/z) were filtered for each metabolite. Peak alignment was performed across samples with a retention time deviation of 0.2 min and a mass deviation of 5 ppm. Peak extraction was then conducted using thresholds of 5 ppm mass deviation, 30% signal intensity deviation, signal-to-noise ratio≥3, minimum signal intensity, and adduct ion information. Peak areas were quantified, and target ions were integrated. Molecular formulas were predicted based on molecular ion peaks and fragment ions, followed by comparison with the mzCloud (https://www.mzcloud.org/), mzVault, and Masslist databases. Background ions were removed using blank samples, and raw quantitative results were normalized to obtain identified metabolites and their relative quantification results. Identified metabolites were annotated using the Kyoto Encyclopedia of Genes and Genomes (KEGG) (https://www.genome.jp/kegg/pathway.html), and LIPID Metabolites & Pathways Strategy (LIPIDMaps) (http://www.lipidmaps.org/) databases.

#### 2.9.2 Targeted data processing

Raw data files (.raw) were imported into Qual Browser software for analysis. Parameters such as retention time and m/z were filtered for each metabolite. Gaussian smoothing was applied, and nine key parameters were set to quantify the peak areas of four alkaloids in chromatograms for each sample. The concentrations of the four alkaloid components in each sample were calculated based on standard calibration curves.

#### 2.9.3 Nutritional element data processing

The contents of mineral elements (K, Na, Mn, Fe, Al, Cu, Mg, Zn) in samples were calculated according to the formula specified in Method 2 of GB 5009.268-2016 (Chinese National Standard). The Formula (8) is expressed as:


(8) $$X=\frac{(C-C_{0})*V*1000*f}{m*1000}$$

where:

*X*–The content of elements to be tested in the sample solution is mg/kg;

*C*–The concentration of the measured element in the sample solution is mg/L;

*C_0_*–The content of the measured elements in the reagent blank solution was mg/L;

*V*–The constant volume mL of sample digestive fluid;

*f*–Sample dilution multiple;

*m*–Sample weight g.

## 3 Results and discussion

### 3.1 Untargeted metabolomic profiling

**Figure 2:**
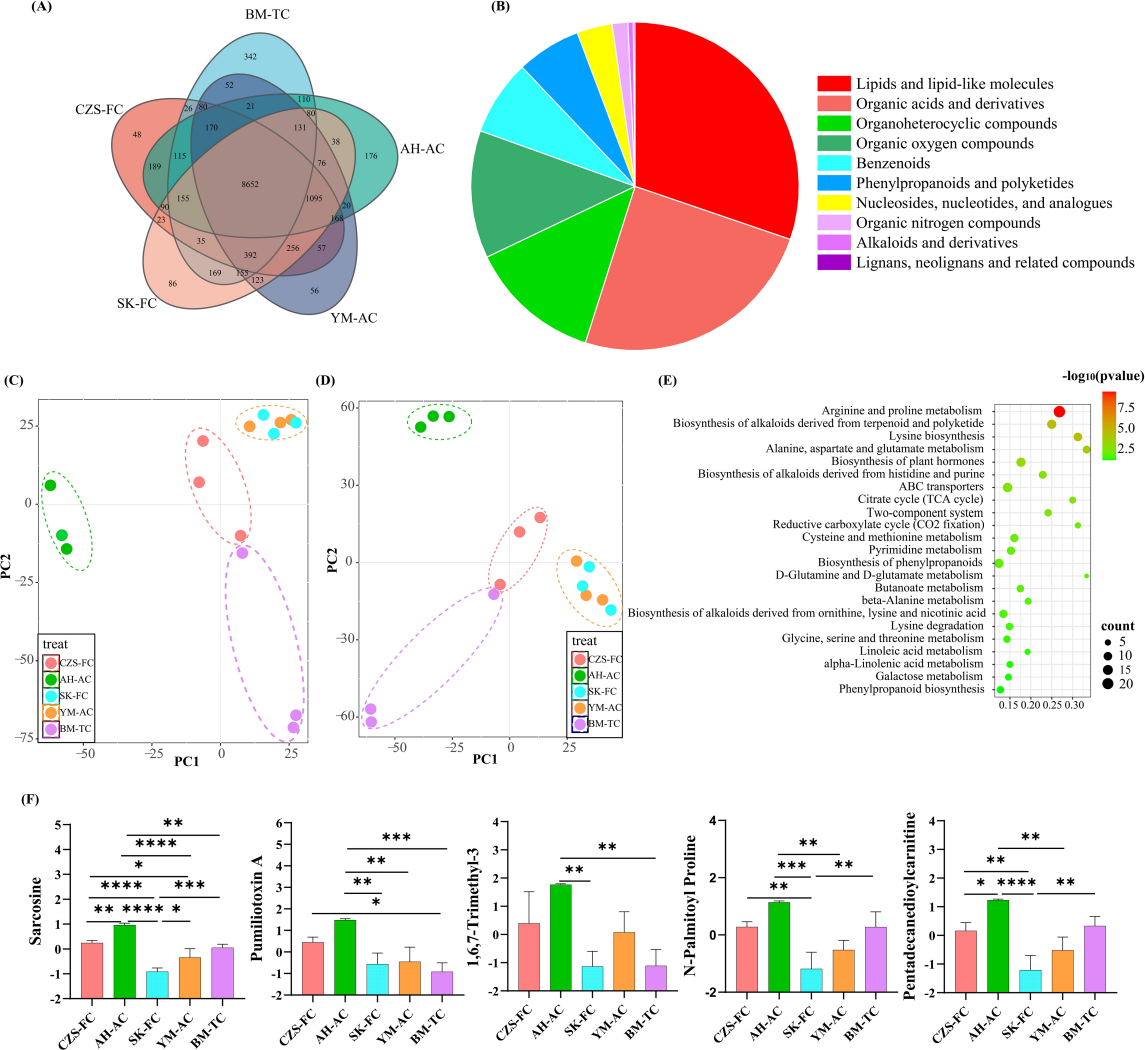
Untargeted metabolomic analysis of FCB. **(A)** Comparative metabolite profiles of FCB from different sources; **(B)** Metabolite category distribution; **(C)** PLS-DA score plot in positive ion mode; **(D)** PLS-DA score plot in negative ion mode; **(E)** KEGG pathway enrichment analysis; **(F)** Content of five key metabolites in FCB from different sources. Asterisks are used to denote statistically significant differences: The significance of the results is indicated by the following p-values: p<0.05, ** p<0.01, p<0.001, and **** p<0.0001, among others.

Ionizable metabolites in FCB from various sources were comprehensively characterized using UPLC-MS, with total ion chromatogram (TIC) and results illustrated in **Figure 2A** and **Supplementary Figure S2A-D**. As shown in the **Figure 2A**, the five different FCB sources shared 8,652 common metabolites, while unique metabolites were identified as follows: 342 in BM-TC, 176 in AH-AC, 56 in YM-AC (Yimu township crtificial cultivated accessions), 86 in SK-FC, and 48 in CZS-FC. **Figure 2B** displays the proportional distribution of metabolite categories. Lipids and lipid-like molecules accounted for the largest proportion (30.25%), followed by organic acids and derivatives (24.63%). Organic heterocyclic compounds and organic oxygen-containing compounds showed similar proportions, at 13.01% and 12.58%, respectively. Benzene-type compounds, phenylpropanoids, and polyketides constituted 7.44% and 6.35%, respectively. Nucleosides, nucleotides and analogues, nitrogen-containing organic compounds, alkaloids and derivatives, lignans, neolignans, and related compounds collectively represented 5.74%. Subsequently, PLS-DA discrimination models were constructed based on metabolites detected in positive ion mode and negative ion mode, with results shown in **Figure 2C, D**. Both models effectively differentiated CZS-FC, AH-AC, and BM-TC samples but failed to distinguish SK-FC and YM-AC, likely due to their shared geographical source (Daofu County and Ganzi Prefecture), resulting in minimal metabolic differences.

Variable Importance in Projection (VIP) analysis of PLS-DA results identified metabolites with VIP > 1 (**Supplementary Table S2**), which were subjected to KEGG pathway enrichment analysis (**Figure 2E**). KEGG enrichment revealed 23 pathways significantly influenced by these differential metabolites, primarily involving arginine and proline metabolism, biosynthesis of terpenoid and polyketide alkaloids, lysine biosynthesis, alanine, aspartate, and glutamate metabolism, phytohormone biosynthesis, and biosynthesis of alkaloids derived from histidine and purine, among others. Finally, the top five key metabolites were selected, and their contents in FCB from different sources were analyzed (**Figure 2F**). AH-AC samples exhibited the highest levels of these metabolites across all sources, likely due to significant differences in soil nutrients and mineral elements between wild and cultivated habitats (Li et al., 2024). CZS-FC samples also showed higher metabolite levels compared to other sources. This phenomenon may be attributed to the fact that both locations are situated within the Songpan region, which is the primary production area for FCB (Lin et al., 2025). Concurrently, it is noteworthy that FCB specimens from disparate origins manifest variable characteristics, which are predominantly manifested in 23 metabolic pathways, encompassing arginine, proline metabolism, and terpenoid biosynthesis (Wang et al., 2025). These pathways may influence FCB’s adaptation to the high-altitude environment, characterized by low temperatures, dryness, and hypoxia, as well as the synthesis of alkaloids and saponins. This finding provides molecular-level evidence for the geographical dependence of FCB’s secondary metabolites. These differential metabolic pathways may affect the synthesis of active components in FCB To test this hypothesis, the present study conducted targeted metabolomics analysis on four key alkaloids.

### 3.2 Targeted analysis results

**Figure 3:**
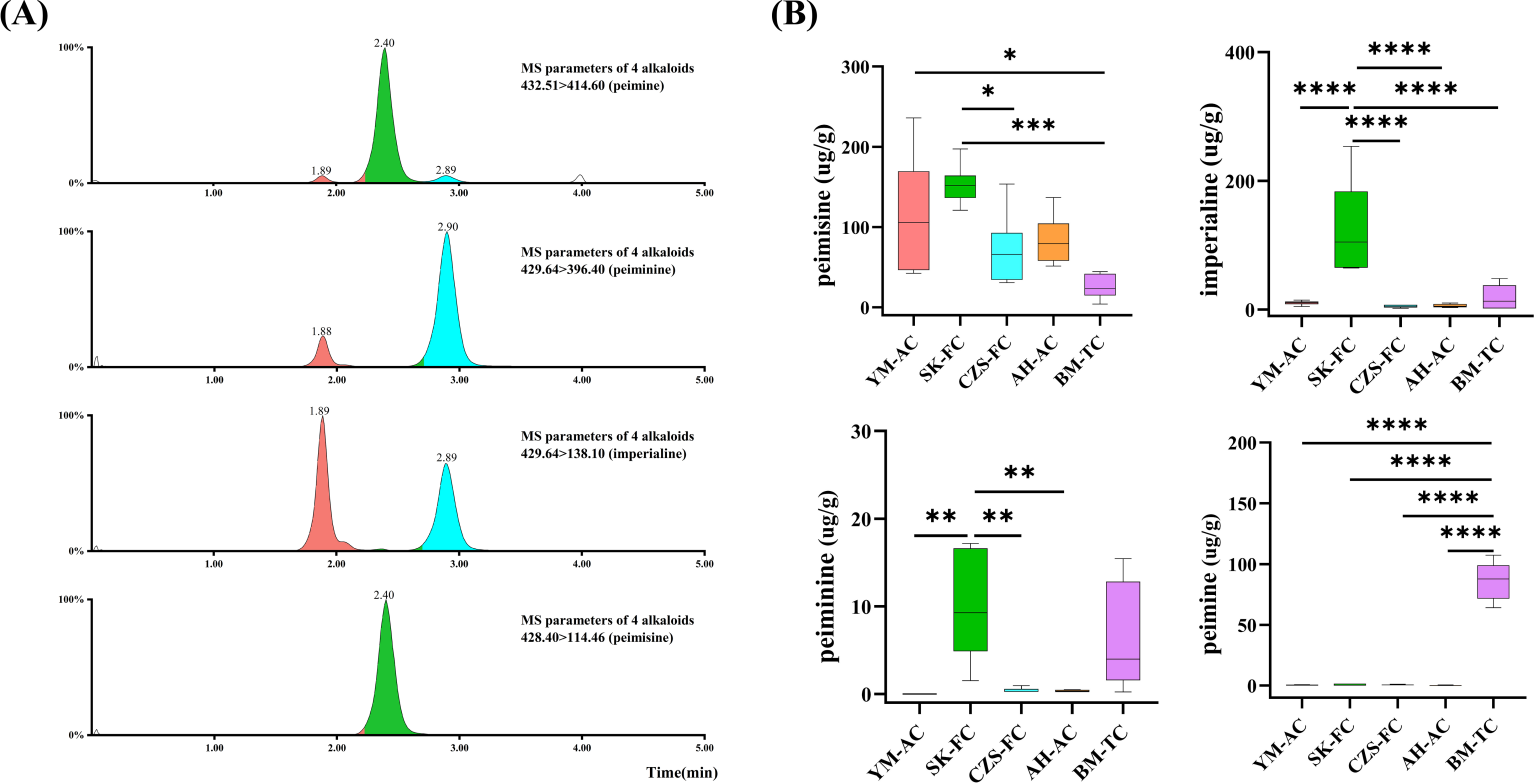
Targeted analysis of FCB. **(A)** Typical multiple reaction monitoring chromatograms of alkaloids in FCB from different sources; **(B)** Content of four alkaloids in FCB from different sources.

Multiple studies have demonstrated that alkaloids are the primary active components responsible for the pharmacological effects of FCB (Chang et al., 2020; Chen et al., 2020; Wang et al., 2016). Therefore, this study quantified the contents of four major alkaloids in FCB from different sources using UPLC-MS/MS. The MS/MS fragmentation patterns of FCB alkaloids are shown in **Figure 3A**, sequentially corresponding to peimisine, imperialine, peiminine, and peimine. Notably, imperialine and peiminine are isomers (C_27_H_43_NO_3_), resulting in two distinct retention time peaks: 1.89 min for imperialine and 2.90 min for peiminine. The alkaloid contents in FCB from different sources, calculated based on standard calibration curves (**Supplementary Table S1**), are illustrated in **Figure 3B**. All five FCB sources contained detectable levels of peimisine and imperialine, while peiminine and peimine were undetectable in some sources, consistent with findings reported by Zhang et al. (2022).

Among the collected samples, SK-FC-derived FCB exhibited significantly higher levels of peimisine (120.69-197.36 µg/g), imperialine (64.71-253.62 µg/g), and peiminine (1.53-17.17 µg/g) compared to other sources. This phenomenon is likely attributed to unique soil nutrients, altitude, and climatic variables in Seka Township, Daofu County, which regulate secondary metabolic pathways and drive high alkaloid accumulation (Chen et al., 2025). Only BM-TC-derived FCB contained elevated levels of peimine (64.13-107.42 µg/g). Although BM-TC samples showed lower peimisine, imperialine, and peiminine contents than SK-FC, they uniquely accumulated peimine, which is absent in wild varieties. Compared to cultivated sources, BM-TC samples displayed higher levels of most alkaloids except peimisine.In addition, Zhao et al. (2018) also demonstrated that tissue culture enhances FCB alkaloid synthesis. The biosynthesis of peimine may be regulated by the type and concentration of carbon sources in the culture medium, leading to its enrichment in BM-TC-derived FCB (Ptak et al., 2020). These findings indicate significant variations in alkaloid profiles across FCB sources, particularly the specific enrichment of peimine in tissue-cultured samples (BM-TC) and the high accumulation of peimisine and imperialine in SK-FC samples. The present study found that tissue culture technology promoted the synthesis of FCB, providing new insights for optimizing cultivation strategies. Subsequent experiments are necessary to substantiate these findings. The present study was conducted with the objective of investigating the potential driving factors behind cultivation environment differences. To this end, a further analysis of the nutrient content in FCB was conducted, with a particular focus on its correlation with alkaloid synthesis.

### 3.3 Results of mineral element accumulation characteristics

**Figure 4:**
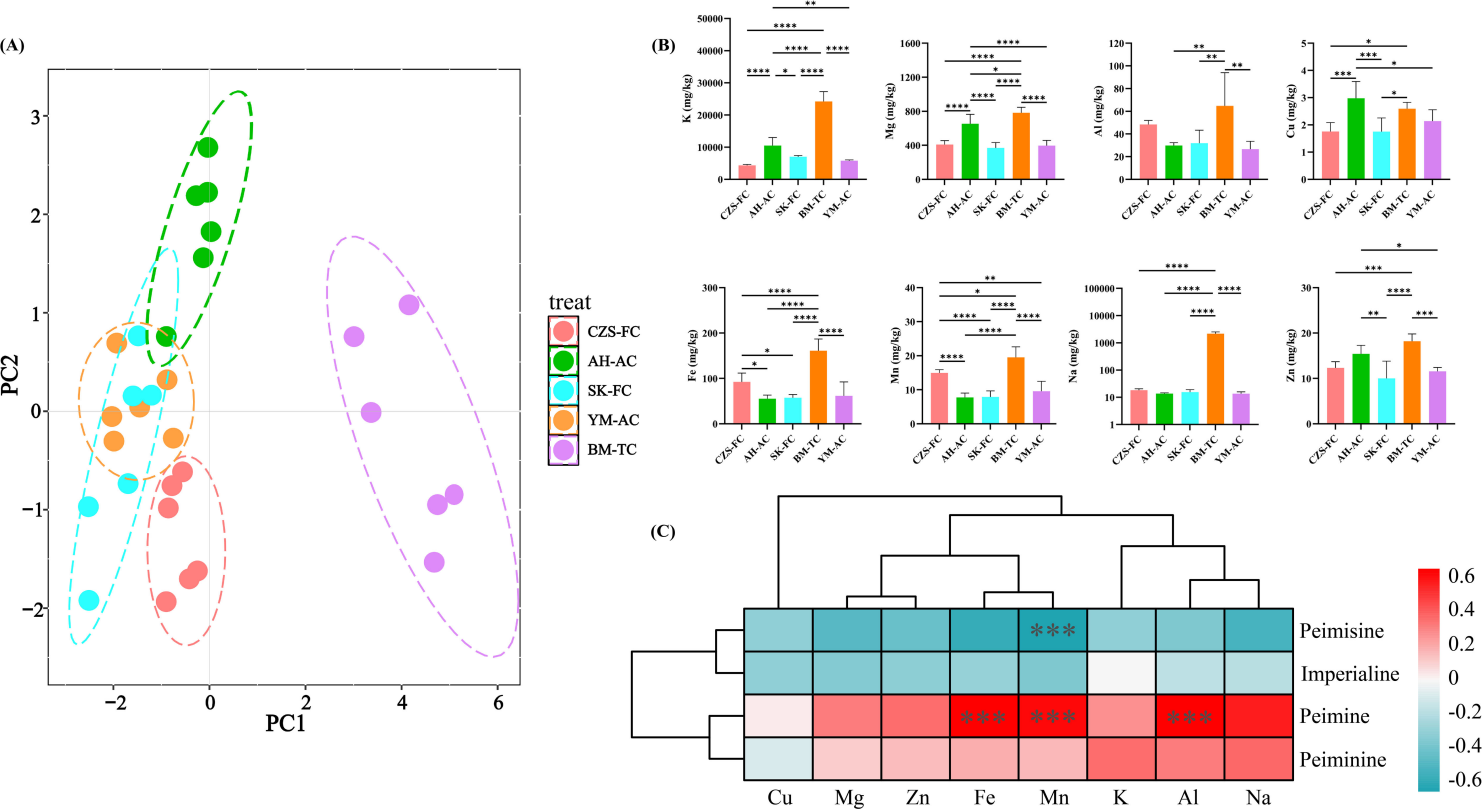
Elemental analysis of FCB. **(A)** PCA plot; **(B)** Content of eight mineral nutritional elements in FCB from different sources; **(C)** Correlation analysis between mineral nutritional elements and alkaloids.

The mineral element contents of FCB from different sources were accurately determined using ICP-MS. Principal component analysis (PCA) was performed on the mineral element data to visualize similarities and differences among samples at the elemental level, with results shown in **Figure 4A**. The PCA plot revealed significant divergence of BM-TC samples from other sources, enabling effective differentiation. CZS-FC, SK-FC, and YM-AC samples exhibited relative proximity but showed no overlap. AH-AC, SK-FC, and YM-AC samples demonstrated partial overlap, indicating some similarity. Notably, AH-AC samples displayed pronounced separation along the positive half-axis of PC2, while some SK-FC samples showed separation trends along the negative half-axis of PC2.

The nutritional element contents of FCB from different sources are illustrated in **Figure 4B**. BM-TC samples exhibited the highest elemental accumulation, likely due to the high concentrations of nutrients and trace elements in the culture medium used for tissue cultivation (Deepika and Singh, 2021). Under laboratory conditions, tissue-cultured FCB may accumulate more nutrients compared to seed-derived plants, as the MS medium used in tissue culture provides elements critical for later developmental stages, enabling continued nutrient absorption post-transplantation. Additionally, phytohormones or chemical additives during cultivation might influence elemental uptake (Tyunin et al., 2020). Further experiments and data analyses are required to clarify the exact mechanisms and optimize cultivation protocols.Apart from BM-TC, CZS-FC samples contained higher levels of Al (43.85-52.94 mg/kg), Fe (70.30-115.85 mg/kg), Mn (13.65-16.06 mg/kg), and Na (15.09-21.63 mg/kg), potentially linked to the native soil background of Songpan County, Aba Prefecture (Song et al., 2022). AH-AC samples showed elevated contents of K (7239.18-13342.10 mg/kg), Mg (518.71-818.63 mg/kg), Cu (2.35-3.68 mg/kg), and Zn (12.86-18.11 mg/kg), likely influenced by fertilizer application in artificial cultivation (Brodowska et al., 2022; Pongrac et al., 2019). Subsequently, correlations between mineral elements and the four major alkaloids in FCB were investigated (**Figure 4C**). As shown in the figure Fe, Mn, and Al exhibited significant positive correlations with peiminine, while Mn showed a significant negative correlation with peimisine. Additionally, most elements demonstrated negative correlations with peimisine and imperialine but positive correlations with peiminine and peimine. Currently, there are relatively few reports on the relationship between nutritional elements and the four main alkaloids in FCB The results of this study establish a foundation for future research on the element-mediated secondary metabolic regulation mechanisms in FCB.

### 3.4 Traceability model results

**Figure 5:**
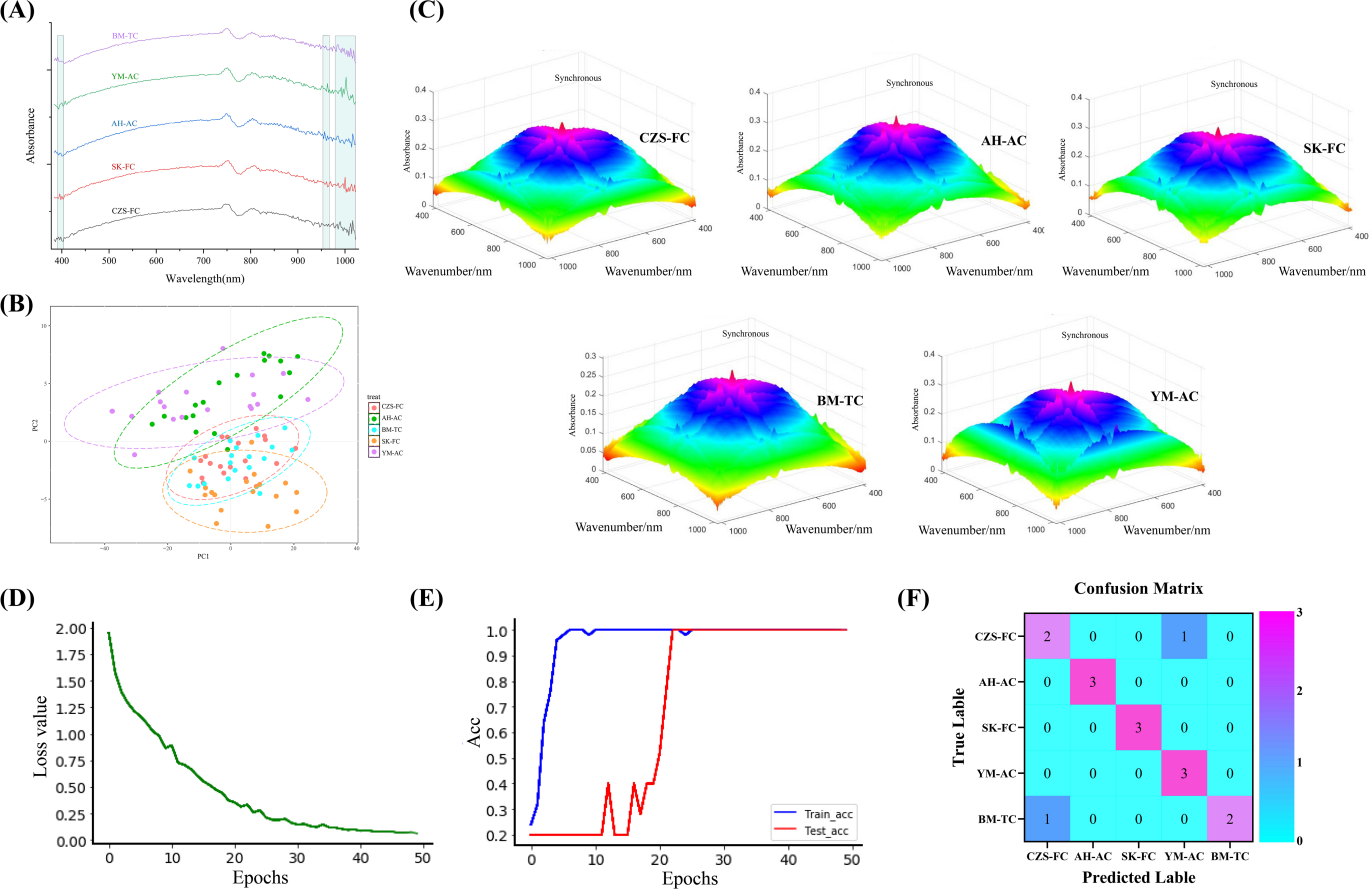
Traceability model results. **(A)** Hyperspectral profiles of FCB from different sources; **(B)** PLS-DA results; **(C)** 3DCOS images of FCB from different sources; **(D)** Model distortion rate; **(E)** Model accuracy rate; **(F)** Confusion matrix.

Multivariate analysis revealed significant differences in the chemical profiles of FBC from different geographical sources. Characteristic wavelength measurements have been demonstrated to be suitable for the quantitative analysis of specific constituents (Hu et al., 2024). Additionally, the heterogeneity of these chemical profiles could be characterized using spectroscopic techniques. **Figure 5A** presents the hyperspectral curves of FBC from diverse geographical origins. Despite the observed variations in reflectance intensity among the spectral curves, a similarity in the overall absorption peak positions was noted. These characteristic absorption peaks correspond to vibrational and stretching modes of functional groups or chemical bonds within the phytochemical constituents (Wei et al., 2024), indicating a high degree of compositional similarity among the samples to a certain extent. As demonstrated in **Supplementary Figure S3A**, the absorption peaks for all samples were predominantly centered at 750, 800, 840, and 970 nm. Among these, the absorption peak near 750 nm may be attributed to the third overtone of carbon-hydrogen (C-H) vibrations in organic compounds, including sugars (e.g., glucose, fructose), organic acids (e.g., tartaric acid), and alcohols (Cui et al., 2025). The absorption band around 800 nm is potentially associated with flavonoid components (Wang S. M. et al., 2022).The absorption feature near 840 nm is likely related to C-H vibrations in sugar-dominated organic compounds within the bulb (Shao et al., 2024). The absorption band observed near 970 nm may be attributed to the second harmonic of O-H vibrations, correlating with sample moisture content (Gao et al., 2022). Alternatively, the observed phenomenon may be associated with the third and second harmonic of O-H vibrations in the chemical components of saponin within this spectral region (Sun et al., 2024).

With respect to samples from disparate origins, comparative analysis of **Supplementary Figure S3A, B** demonstrated that the BM-TC sample exhibited diminished overall values across the 460-750 nm range in comparison to other samples. This phenomenon can be attributed to the comparatively brief growth period of the tissue culture cultivation (BM-TC) relative to the seeding samples. This relative paucity of growth time may result in lower concentrations of chemical constituents, including saponins, flavonoids, and polysaccharides, within this particular wavelength range (Hu et al., 2024). This finding further underscores the influence of sufficient cultivation duration on the chemical composition of FCB derived from tissue culture cultivation. Concurrently, the BM-TC and AH-AC samples exhibited a high degree of similarity below 460 nm, suggesting that artificially cultivated samples share certain compositional similarities. In contrast, the hyperspectral curves of the four seedling-derived FCB samples exhibited a high degree of consistency beyond 460 nm.

In general, hyperspectral technology can effectively distinguish between cultivated and wild products. Hu et al. (2023) also found similar results in their research. Hyperspectral spectra of wild FCB *fr*om different sources show slight differences, making it difficult to accurately identify them by visual observation alone. To address this, this study applied machine learning techniques to analyze hyperspectral data from five FCB sources, aiming to achieve traceability of FCB origins.

This study first performed dimensionality reduction analysis on the hyperspectral dataset of FCB and constructed a PLS-DA model, with results shown in **Figure 5B**. The results revealed extensive overlap in the identification regions of the five FCB sources, with some samples remaining unclassified, indicating the inability of traditional PLS-DA to distinguish FCB origins. To overcome these limitations, deep learning methods were introduced to develop a traceability model.

Compared to traditional machine learning models, the identification system combining visible-near-infrared spectroscopy with deep learning models eliminates the need for complex preprocessing while achieving higher accuracy and superior generalization capability, demonstrating greater precision and robustness (Wang et al., 2024b). The study produced 3DCOS spectra, as demonstrated in **Figure 5C**, derived from hyperspectral datasets originating from various sources of FCB These datasets were subsequently employed for the ResNet modeling process.The relationships between Acc/Loss value and epochs are depicted in **Figure 5D, E**, respectively, while the external validation results are shown in **Figure 5F**.

As shown in the above figure, when the iteration number reached 22, the ResNet model achieved 100% accuracy for both the training and testing sets, with a loss value of 0.334. Among the 15 samples in the external validation set, 13 were correctly classified, while 2 samples were misclassified, yielding an accuracy of 86.667%, demonstrating robust classification performance. The two classification errors involved a CZS-FC sample misclassified as YM-AC and a BM-TC sample misclassified as CZS-FC. These errors may arise from the fact that all samples were collected from Sichuan Province, China, where some samples exhibit minor differences in chemical composition. Additionally, the limited sample size may have impacted the model’s generalization capability, leading to classification errors in the external validation set.

Based on these results, the ResNet model constructed using 3DOCS images in this study excels across multiple performance metrics, achieving high accuracy. It also exhibits strong generalization ability, enabling precise identification of FCB feature differences under varying conditions and thereby realizing high-accuracy traceability of FCB from different sources.The multidimensional analysis results revealed significant differences in the chemical characteristics of FCB from different sources. The chemical heterogeneity can be characterized using spectroscopic techniques. Consequently, this study employed hyperspectral data to construct 3DCOS images, thereby capturing the microscopic chemical fingerprints of FCB. The integration of 3DCOS images with the ResNet deep learning model resulted in the establishment of a traceability model that distinguishes FCB from different origins. The experimental results demonstrated that the model achieved 100% accuracy on the testing set and 86.67% accuracy on the validation set, thereby significantly outperforming traditional PLS-DA methods. Although classification errors occurred for a few samples in external validation due to geographical proximity or chemical similarity, the model’s overall performance highlights its high reliability and generalization potential for FCB traceability. This study corroborates the prospective implementation of spectral technology in conjunction with multivariate data processing methodologies for the assessment of quality disparities in TCM (Li et al., 2023; Wu et al., 2023; Qi et al., 2024).

## 4 Conclusion

This study revealed the significant effects of geographical origins and cultivation methods on the metabolite composition, alkaloid content, and mineral element accumulation in FCB through multidimensional analyses, thereby establishing a “metabolism-component-environment” multidimensional evaluation framework. The results demonstrate that FCB quality characteristics exhibit significant heterogeneity due to environmental factors such as soil nutrients, climatic conditions, and cultivation practices. The ResNet deep learning model, constructed using hyperspectral-derived 3DCOS images, achieved 100% testing/validation accuracy and 86.67% external validation accuracy, providing an efficient method for precise FCB traceability. Integrating multidimensional analysis with deep learning establishes the theoretical foundations for FCB quality evaluation and origin tracing, offering fundamental support for further development and utilization of FCB resources. To enhance the generalizability and translational potential of the model, future research should prioritize the following: (1) Cross-regional validation: The expansion of sampling to encompass core production zones, including Yunnan, Tibet and Qinghai, among others, is imperative for the execution of large-scale cohort studies. This expansion is necessary to optimize parameters and validate geographical transferability. (2) Integrated intelligent tracing: Developing a tripartite decision-making model (“chemical fingerprint-environmental imprint-agronomic indicators”) by combining hyperspectral imaging with key environmental covariates (elevation gradients, soil micronutrient profiles) and cultivation practices to improve robustness in complex ecosystems; (3) Methodology transfer: The multidimensional 3DCOS-ResNet framework is applied to geographically sensitive medicinal materials to establish standardized quality traceability protocols.

## Data Availability

The original contributions presented in the study are included in the article/**Supplementary Material**. Further inquiries can be directed to the corresponding authors.
