# Supplementary material for: Multidimensional quality evaluation and traceability study of Fritillariae Cirrhosae Bulbus from different sources
Source: Front Plant Sci. 2025 Sep 11;16:1648434. doi: 10.3389/fpls.2025.1648434 (PMC12460310; doi:10.3389/fpls.2025.1648434)
Supplement: Supplementary file 4 [file Table1.docx]

**Table S1** Standard curve , correlation coelficients( r^2^), range, LODs , LOOs , recoveries and of 4 alkaioids

| Component | Standard curve | r^2^ | Range (μg/mL) | LODs（ng/mL） | LOQs（ng /mL） | Inter-day (%) | Intra-day (%) | Average recovery rate(%) , n=3 |
| --- | --- | --- | --- | --- | --- | --- | --- | --- |
| peimisine | y=7,530.65x-2922.56 | 0.9991 | 0.001-0.200 | 0.030 | 0.100 | 2.73 | 3.92 | 90.60 |
| imperialine | y=8,910.56x-3,606.90 | 0.9983 | 0.001-0.200 | 0.006 | 0.020 | 3.47 | 4.85 | 96.03 |
| peiminine | y=41,787.5x-18,537.6 | 0.9992 | 0.001-0.200 | 0.004 | 0.010 | 1.08 | 3.34 | 92.80 |
| peimine | y=244,53.6x-12,622.7 | 0.9994 | 0.001-0.200 | 0.003 | 0.010 | 2.52 | 4.17 | 97.63 |

**Table S2** Microwave digestion conditions

| method to dispel | procedure | Control temperature (℃) | climbing time (min) | holding time(min) |
| --- | --- | --- | --- | --- |
| microwave digestion | 1 | 120 | 5 | 10 |
|  | 2 | 150 | 5 | 20 |
|  | 3 | 190 | 5 | 30 |
